# A 7.5-mV Input and 88%-Efficiency Single-Inductor Boost Converter with Self-Startup and MPPT for Thermoelectric Energy Harvesting

**DOI:** 10.3390/mi14010060

**Published:** 2022-12-26

**Authors:** Chuting Wu, Jiabao Zhang, Yuting Zhang, Yanhan Zeng

**Affiliations:** School of Electronics and Communication Engineering, Guangzhou University, Guangzhou 510006, China

**Keywords:** thermoelectric energy harvesting, maximum power point tracking, boost converter, self-startup

## Abstract

This paper presents a single-inductor boost converter for thermoelectric energy harvesting. A two-stages startup circuit with a three-phase operation is adopted to obtain self-startup with a single inductor. To extract the maximum energy, a coarse- and fine-tuning MPPT is proposed to adaptively and effectively track the internal source resistance. By designing a zero-current detector, the synchronization loss is reduced, which significantly improves the peak efficiency. The boost converter is implemented in a 0.18-μm standard CMOS process. Simulation results show that the converter self-starts the operation from a TEG voltage of 128 mV and achieves 88% peak efficiency, providing a maximum output power of 3.78 mW. The improved MPPT enables the converter to sustain the operation at an input voltage as low as 7.5 mV after self-startup.

## 1. Introduction

Energy harvesting technology can be used in passing sensing devices in internet of things (IoTs) [1,2,3] due to the self-power [4,5], which can provide the supply voltage instead of the battery. Recently, there is a lot of research concentrating on harvesting piezoelectric energy [6], radio frequency (RF) energy [7], photovoltaic energy [8] and thermoelectric energy [9,10,11]. Among them, thermal energy can be widely used in wearable devices [12] because the thermoelectric generator (TEG) can convert the temperature difference between human body and the environment into a voltage. However, the output voltage from TEG is as low as tens of millivolts when the temperature difference is ultra-low. Thus a boost converter is needed in the thermal energy harvesting system.

In recent years, several boost dc–dc converters have been reported for low power operation. Ref. [13] proposed a converter with a zero current switching (ZCS), which operates at the input voltages ranging from 20 mV to 250 mV. However, there is no MPPT circuit to extract the maximum energy and the startup process is completed by an additional source. Meanwhile, a MPPT techinique for variation tolerance was proposed in [14] to improve the overall efficiency, but the converter utilizes a battery to startup. Boost converters such as [15,16] can lower the input voltage to tens of millivolts. However, these converters fail to self-start from a low TEG voltage, which is unable to realize automatic operation.

To achieve coldstart, ref. [17] uses an off-chip transformer but increases the costs and sizes. The transformer-reuse technique restricts the peak efficiency. A fully integrated startup approach is adopted in [18,19]. However, the peak efficiency is only 58% and 76%, respectively, due to the improper loss control. Ref. [20] achieves a high peak efficiency of 83%, but it only starts from a TEG voltage of 220 mV, which is not low enough. Therefore, it is challenging to achieve a lower voltage startup and high efficiency with only one inductor.

This paper proposes a 88%-efficiency single-inductor boost converter for low power thermoelectric energy harvesting. A two-stage startup circuit with three phase operation is proposed to assist in self-startup of 128 mV. An accurate MPPT is proposed to ensure the maximum power harvesting even in a low input voltage of 7.5 mV, which, together with the improved zero current detector (ZCD), also can improve the end-to-end efficiency within a wide load range.

This paper is organized as follows. The principle of the proposed converter is introduced in Section 2. In Section 3, the concrete circuit implementation is presented. Section 4 shows the verification results, and the conclusion is drawn in Section 5.

## 2. The Principle of Proposed Converter

**Figure 1:**
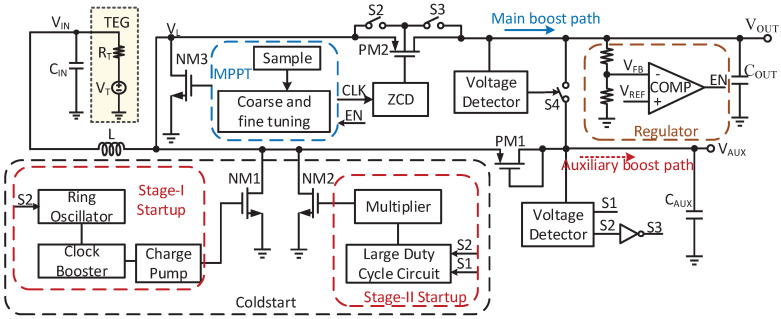
Architecture of the proposed converter.

As shown in Figure 1, a dual-path boost converter using a single inductor is proposed. It mainly consists of several parts including the coldstart, MPPT, ZCD, voltage detectors and regulator. The proposed converter starts its operation from only 128 mV by the two-stage startup circuit. The stage-I startup circuit consists of the ring oscillator, clock booster and charge pump. The large duty cycle circuit and 3-stage multiplier are utilized in the stage-II startup.

### 2.1. Proposed Three Phase Operation

**Figure 2:**
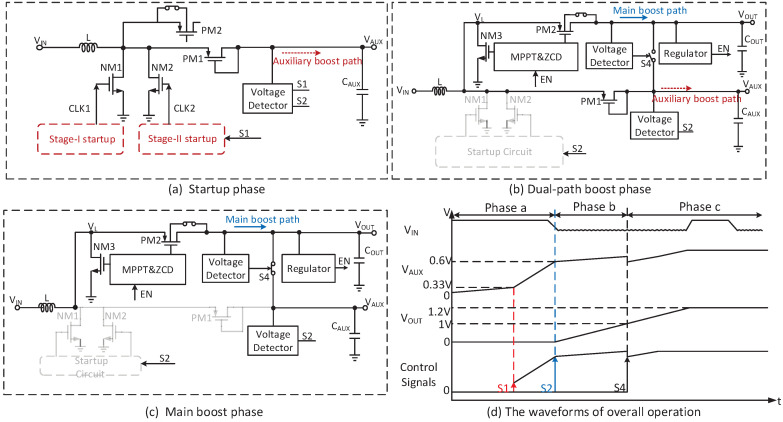
The three phases of the converter.

One of the challenges of this design is to support the low coldstart voltage while entering the normal operation. In previous cold-start works, the switched capacitor voltage multiplier, charge pump and voltage multiplier are usually utilized to improve the conversion efficiency for the initial boost, but their output power is low, which is not suitable for the main converter operation. However, none of the above converters meet the requirements of low input voltage, fast start boost and high conversion efficiency simultaneously. In this paper, a two-stage startup circuit with three phases is proposed to overcome the compromise between the low input voltage and efficiency for the TEG application. These output voltage levels are determined by the signal S2 and S4 from the voltage detectors. The illustration of the whole operation is presented in Figure 2.

In the startup phase, the stage-I startup self-starts from TEG supply and drives NM1, as shown in Figure 2a. Since the converter is weak, the load in this phase should be minimized. The ultra-low-power voltage detectors are designed, which is presented in Section 3.3.

In order to accumulate energy and generate a sufficiently high voltage at $V_{AUX}$, $C_{AUX}$ is designed to be small enough (1 nF). Once $V_{AUX}$ is higher than 330 mV, the stage-II startup is activated by S1 to drive NM2. The charging on $C_{AUX}$ remains until $V_{AUX}$ reaches 600 mV to trigger S2. The bulk of PM2 is biased to $V_{L}$ during the self-starting, which avoids the insufficient current to the body diode. However, since $V_{OUT}$ is not generated yet, a dual-path boost phase is needed to assist the converter to enter normal operation.

In the dual-path boost phase, the control modules are supplied by $V_{AUX}$. MPPT and ZCD are enabled by S2 to provide the clock for NM3 and PM2, as shown in Figure 2b. However, PM1 is a diode structure which causes a drop-off loss. Once $V_{OUT}$ reaches 1 V, $V_{AUX}$ and $V_{OUT}$ are shortened by S4.

In the main boost phase, $V_{OUT}$ supplies the control modules so that the converter can work independently, as shown in Figure 2c. Finally, the converter works in a steady state. NM3 and PM2 are temporarily turned off by EN once $V_{OUT}$ exceeds 1.2 V. Both in the dual-path boost and main boost phase, the bulk of PM2 is biased to $V_{OUT}$, which can prevent the output current from reversing to the body diode. Figure 2d shows the waveforms of the whole operation.

The proposed dual-path converter adopts three phases to complete the coldstart and ultimately enters high-efficiency mode without an additional inductor.

### 2.2. Efficiency Improvement

Another challenge is to improve the efficiency in normal operation. In this paper, the coldstart block is fully turned off to avoid the additional power consumption. To provide as much power as possible to the load with sufficiently high output voltage, the converter should first extract the maximum power from TEG, then efficiently transfer the power to the load. Owing to the input as low as tens of millivolts, the discontinuous conduction mode (DCM) is suitable for the converter to reduce switching loss.

#### 2.2.1. Tracking Efficiency

To obtain the maximum input power, it is important to select the value of the inductor, the on-time of NM3, and the switching frequency. In DCM, the average inductor current can be expressed as

(1) $$I_{AVE}=\frac{V_{IN}t_{LS}^{2}}{2L\times T},$$

where *L* is the value of inductor, $t_{LS}$ is the on-time of NM3 and *T* is the working period. Therefore, the extracted input power is obtained as

(2) $$P_{IN,ave}=V_{IN}I_{AVE}=\frac{V_{IN}^{2}t_{LS}^{2}}{2L\times T}.$$

When the converter operates in the maximum power point (MPP), $V_{IN}$ is equal to $V_{T}$/2. $P_{IN,ave}$ can be also expressed as

(3) $$P_{IN,ave}=\frac{V_{T}^{2}t_{LS}^{2}}{8L\times T}=\frac{V_{T}^{2}t_{LS}\times D}{8L},$$

where *D* is the duty cycle of NM3, which is lower than 1. $P_{IN,MAX}$ is then given as

(4) $$P_{IN,MAX}=\frac{V_{T}^{2}}{4R_{T}}.$$

Therefore, for a given MPP, the value of $t_{LS}$ is depended on *L*. Large *L* requires large $t_{LS}$. However, large $t_{LS}$ causes large ripple in $V_{IN}$, which decreases the tracking efficiency. Contrarily, small *L* needs low $t_{LS}$. The large duty cycle is required to transfer enough power to the load. Therefore, the switching frequency should be large, which increases the switching loss in the low input power. Considering the factors above, a 100 μH inductor is used to extract the maximum power and reduce power loss.

The fractional open circuit voltage (FOCV) method is adopted because of its convenience. In the MPPT mode, the internal resistor of the converter can be expressed as [16]

(5) $$R_{IN}=\frac{2L}{t_{LS}^{2}f}.$$

From this equation, $t_{LS}$ or *f* can be adjusted to track MPP. Considering the efficiency in ultra-low input power, *f* is fixed and $t_{LS}$ is adjusted in this paper.

**Figure 3:**
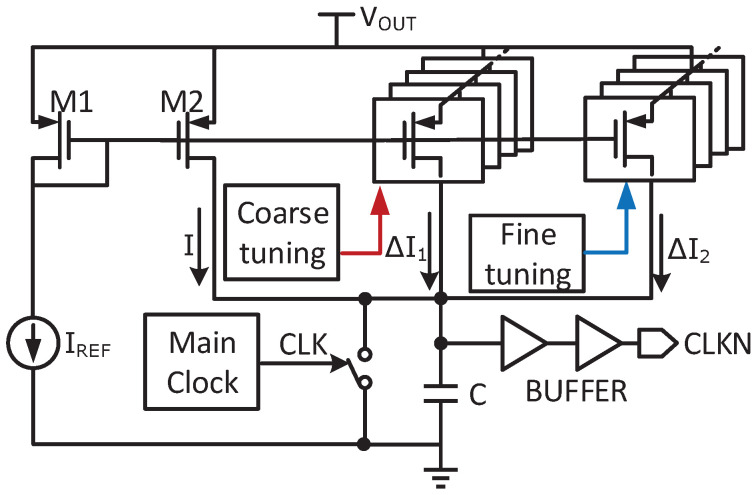
The three phases of the converter.

As shown in Figure 3, $t_{LS}$ is adjusted by the charging current. When $R_{IN}$ is larger than $R_{T}$, $V_{IN}$ is higher than $V_{T}$/2. Then $t_{LS}$ raises to decrease $R_{IN}$ for the impendence matching. Contrarily, $t_{LS}$ can be reduced to increase $R_{IN}$. Because $R_{T}$ depends on the temperature difference of TEG, the traceable $R_{IN}$ should cover a wide range to keep MPP when $R_{T}$ deviates from the design value by 12% [14]. To ensure a high tracking speed, coarse tuning is needed. However, coarse tuning leads to oscillation near MPP, which causes a large ripple in $V_{IN}$. Considering the boost ratio, $R_{IN}$ can be also expressed as

(6) $$R_{IN}=\frac{V_{IN}}{I_{L,ave}}=\frac{2L}{t_{LS}}\left(\frac{1}{1-\frac{V_{IN}}{V_{OUT}}}\right).$$

A small change of $t_{LS}$ may decrease the boost ratio. To overcome these shortages, a fine-coarse tuning is proposed in this design, as shown in Figure 3. In this structure, $t_{LS}$ can be expressed as

(7) $$t_{LS}=\frac{CV_{th}}{I+\Delta I_{1}+\Delta I_{2}},$$

where $V_{th}$ is the threshold voltage of inverter. The range of $R_{IN}$ is defined as $R_{IN1}$, which can be expressed as

(8) $$R_{IN1}=\frac{2L{\left(I+\Delta I_{1}+\Delta I_{2}\right)}^{2}}{{\left(CV_{th}\right)}^{2}f},$$

where $\Delta I_{1}$ is the current change in coarse tuning and $\Delta I_{2}$ in fine tuning. To reduce the power loss in the open-circuit process, the sample period further decreases and less than the switching period. Since the input ripples may cause a deviation in MPP, which lowers the tracking efficiency, the input capacitor is added. The circuit implementation of MPPT is described in Section 3.2 in detail.

#### 2.2.2. Conversion Efficiency

The conversion losses consist of conduction loss, switching loss, synchronization loss and power consumption of the control block [15]. When the input power is low, the switching loss is dominant, which can be expressed as

(9) $$P_{sw}=\left(K_{LS}C_{LS}+K_{HS}C_{HS}\right)fV_{OUT}^{2},$$

where $C_{LS}$ and $C_{HS}$ are the gate capacitors of NM3 and PM2, respectively. $K_{LS}$ and $K_{HS}$ are the power consumption factor of the drivers, respectively, both of which are larger than 1. According to Equation (9), the switching loss remains unchanged when *f* is fixed in DCM.

When the input power is high, the dominant part is conduction loss. Since the on-time of PM2 can be neglected, the conduction loss can be expressed as

(10) $$P_{cond}=f\int_{0}^{t_{LS}}I_{L}^{2}R_{LS}\;dt,$$

where $R_{LS}$ is the on-resistance of NM3 and $I_{L}$ is the inductor current. When the converter works in the MPP mode, $P_{cond}$ is derived as

(11) $$P_{cond}=\frac{{V_{T}}^{2}R_{LS}t_{LS}}{6LR_{T}}.$$

The conduction loss versus the maximum available power can be calculated:

(12) $$\frac{P_{cond}}{P_{IN,MAX}}=\frac{2R_{LS}t_{LS}}{3L}$$

Equation (12) indicates that when $R_{LS}$ is defined, the effect of $P_{cond}$ on the conversion efficiency remains constant while $V_{T}$ changes. To reduce $R_{LS}$, a large NM3 is used. To reduce the conflict between MPPT control and regulation, a burst control utilizing a dynamic comparator is adopted to reduce the conduction loss at light load.

**Figure 4:**
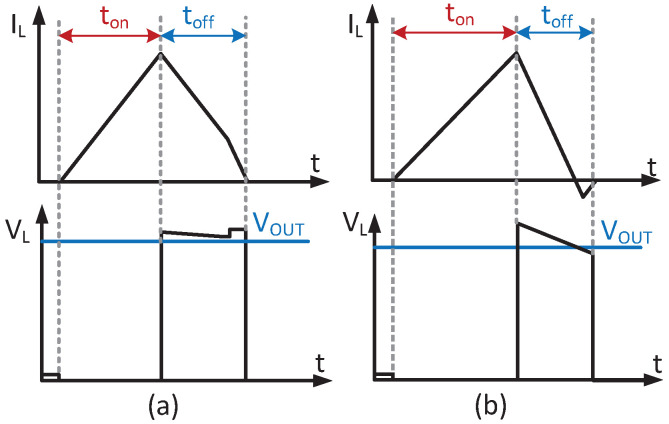
Detailed diagrams when (**a**) PM2 turns off early; (**b**) PM2 turns off late.

Figure 4 shows the non-ideal delay of the PM2 switch. If the PM2 turns off early, a large overshoot will generate in $V_{L}$ as shown in Figure 4a, which is a reduction in conversion efficiency. On the contrary, if PM2 turns off late, the output current will reverse to $V_{L}$ as shown in Figure 4b, which also results in a large loss. Meanwhile, the ideal voltage conversion for the boost converter can be calculated as

(13) $$V_{OUT}=V_{IN}\left(1+\frac{t_{ON}}{t_{OFF}}\right)$$

It can be shown that for a high conversion ratio, $t_{OFF}$ becomes very short, which causes a large loss when ZCD is not precisely designed.

To solve these issues, a static comparator with high speed and precision is adopted to control the turn-off time of PM2 dynamically. Meanwhile, the improper deadtime may cause a large shoot-through the current to damage the transistor, which must be controlled reasonably.

In this design, the following measures are taken to reduce the power consumption of control block. To ensure startup at a low input, the ultra-low-power voltage detectors are used. Meanwhile, because the synchronization loss leads to a low conversion efficiency, an accurate ZCD is needed in this paper, which consumes high power. This paper only enables ZCD within the off-time of NM3 to reduce the dynamic power consumption, which is described in Section 3.3.

## 3. Circuit Implementation

### 3.1. Low Voltage Startup

**Figure 5:**
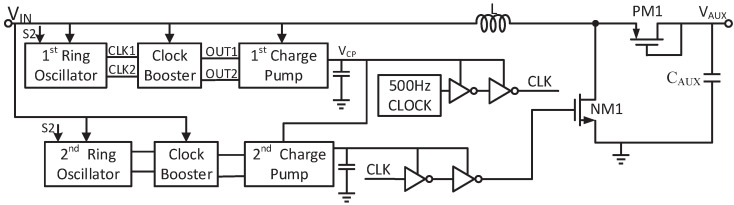
Stage-I startup circuit.

**Figure 6:**
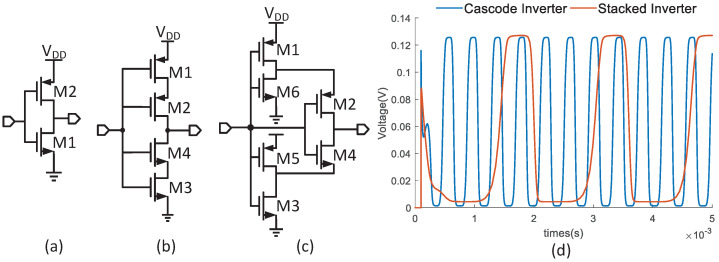
(**a**) Conventional inverter; (**b**) cascode inverter; (**c**) stacked inverter; (**d**) the oscillation of two ring oscillators.

The coldstart is made up of the two-stages startup circuit. The proposed inductor-based stage-I startup circuit is depicted in Figure 5. It consists of ring oscillators, clock boosters and pelliconi charge pumps [21]. Due to the low supply voltage, the output power of the two charge pumps is limited in the pW level. Therefore, the clock used to drive NM1 is designed to be as low as 500 Hz. It is challenging for the system to produce a sustained clock for the charge pumps with a low supply voltage. A feasible scheme is the inverter-based oscillator. Figure 6 shows three kinds of inverters for the ring oscillators. The dc gain in the traditional inverter stage as shown in Figure 6a is given as

(14) $$A_{0}\geq \sqrt{1+{\left(\omega_{1}/\omega_{0}\right)}^{2}},$$

where $\omega_{1}$ is the oscillation frequency and $\omega_{0}$ is the 3 dB bandwidth of the inverter. A low supply voltage degrades the dc gain and the output swing of the inverter. It needs a lot of inverters, which results in low output frequency. The cascode inverter shown in Figure 6b and stacked inverter [22] depicted in Figure 6c can improve the dc gain and the output swing by dynamically reducing the leakage current in the discharging phase. The dc gain of the two inverters can be expressed:

(15) $$A=\frac{\left(1+\frac{g_{m1}}{g_{ds1}}\right)g_{m2}+\left(1+\frac{g_{m3}}{g_{ds3}}\right)g_{m4}}{g_{ds2}+g_{ds4}}$$

where $g_{m}$ and $g_{ds}$ are the transconductance and channel conductance, respectively. The increased dc gain enhances the oscillation capability. However, as shown in Figure 6d, the frequency of the stacked-inverter-based oscillator is much smaller than another one, which lowers the output current of the charge pump. Therefore, this paper uses five cascode inverter stages to build the ring oscillator. The simulated frequency is 2.86 kHz at 128 mV supply.

**Figure 7:**
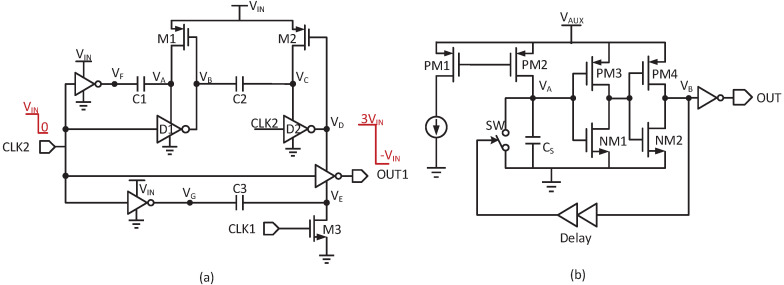
(**a**) The clock booster; (**b**) the large duty cycle circuit.

As shown in Figure 7a, the clock booster with a higher amplitude is used to improve the charge–transfer ability. Once CLK2 is high, $V_{F}$ and $V_{B}$ step low while $V_{C}$ and $V_{A}$ are high. Hence, both C1 and C2 store a voltage of $V_{IN}$. Contrarily, C1 and C2 are connected in series, and $V_{D}$ is boosted to $3V_{IN}$. Similarly, since CLK1 is opposite to CLK2, $V_{E}$ can obtain $-V_{IN}$ when CLK2 is high. In this way, the clock amplitude is boosted to $4V_{IN}$. However, due to the loss and leakage current, the practical swing is smaller than $4V_{IN}$.

**Figure 8:**
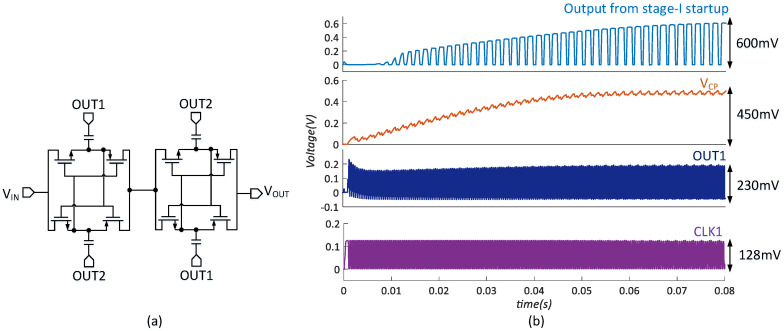
(**a**) The structure of each charge pump; (**b**) stage-I startup simulation waveform when $V_{IN}$ = 128 mV.

In stage-I startup, the two charge pumps are driven by the clocks OUT1 and OUT2 from the clock boosters, which is shown in Figure 8a. If the loss and load are zero, each charge pump could produce an output voltage at 9 times of $V_{IN}$. However, at a low supply, due to the poor clock driving capacity and the leakage current, the charge pump generates a lower boost ratio. Figure 8b shows the simulated results of stage-I startup. The 1st charge pump is driven to charge up $V_{CP}$ to 450 mV with a 100 pA load. The boost ratio is limited to 3.5. Meanwhile, the boost ratio of the 2nd charge pump is limited to 1.5 with a 50 pA load. The efficiency of each charge pump is 4%. Finally, a clock with a 600 mV output swing is obtained.

The stage-II startup consists of the large duty cycle circuit and 3-stage multiplier, which is used to drive NM2. As shown in Figure 7b, when the large duty cycle circuit is activated, $C_{S}$ is charged until $V_{A}$ reaches the threshold voltage of the inverter. Then $V_{B}$ goes high and turns on SW immediately to discharge $C_{S}$. Therefore, $V_{B}$ is set to be a narrow pulse, and the output is a large duty cycle. Since the amplitude is not sufficient to drive NM2, the 3-stage multiplier is used to generate a 4-times output swing.

### 3.2. Accurate MPPT

**Figure 9:**
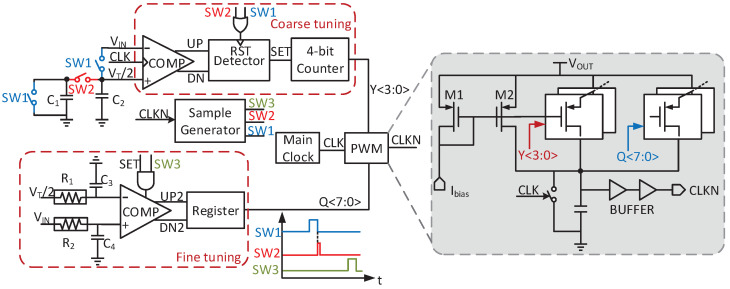
Schematic of the MPPT controller.

Figure 9 shows the structure of the MPPT circuit. Considering the PVT variation, the duty of LS switch is adjusted by controlling the charging current. Once SW1 and SW2 are activated, $V_{T}$ is sampled by disconnecting the converter from the input, and divided in half by the charge sharing of $C_{1}$ and $C_{2}$. The sampling frequency is set to be as low as 1/4096 of the switching frequency. Therefore, the energy loss in the open-circuit process can be neglected.

Then, $V_{IN}$ is compared with $V_{T}$/2 to decide the duty. The coarse- and fine-tuning approach is adopted to obtain accurately and fast tracking. The dynamic comparator and 4-bit counter realize coarse tuning cycle by cycle, whereas the static comparator together with registers starts fine tuning once the coarse tuning is done. Therefore, the duty could be calibrated after several cycles to obtain the maximum energy. The ending signal of the coarse tuning is generated by the detector, which consists of two D flip-flops. When the UP or DN changes, the detector can sense this transition and set the signal SET to high.

A binary-weighted control with a step of 1 μs is employed in the coarse-tuning process. In fine-tuning, the static comparator controls two registers so that increments and decrements can be detected. One register increases the duty with Q<3:0>, while the other decreases the duty with Q<7:4>. Q<7:0> can control the duty cycle with a step of 200 ns.

The quiescent current of the static comparator is 200 nA. To further reduce the average power consumption, the static comparator only works when both SET and SW3 are simultaneously high. Since the internal resistor changes with the charging/discharging action, the ripples of $V_{IN}$ exist, which lowers the accuracy of MPPT. Therefore, a low-pass filter is added to reduce this ripple in fine tuning. The off-chip resistors can be replaced by the pseudo-resistors, which is about 3.4 GΩ in 15-300 mV open-circuit voltage. Both $C_{3}$ and $C_{4}$ are 100 fF. Thus, the cutoff frequency is designed to be about 468 Hz. The simulated peak tracking efficiency is 99% with high accuracy and speed, consuming low power.

### 3.3. Zero Current Detector and Low Power Voltage Detector

**Figure 10:**
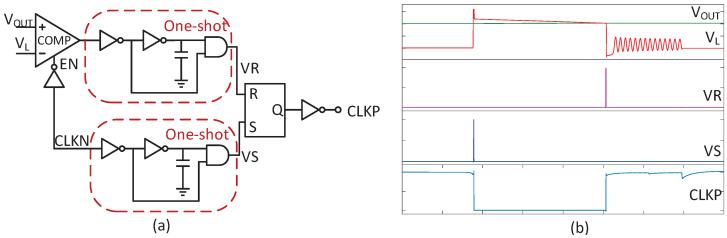
(**a**) The ZCD; (**b**) Simulated waveform of ZCD.

The schematic of ZCD is shown in Figure 10a. It is composed of two one-shot circuits, a RS flip-flop and a static high-precision comparator from [23]. The voltages $V_{L}$ and $V_{OUT}$ are sensed by the comparator per cycle to detect the inductor current direction. Once the difference of two voltage-nodes decreases to 0, the HS switch is turned off immediately. According to Section 3, a proper dead time is needed and realized by the below one-shot circuit. For quickly and accurately tracking, the comparator consumes 6 μA quiescent current. Hence, the comparator is only enabled within the off-time of NM3 to reduce the average power consumption.

**Figure 11:**
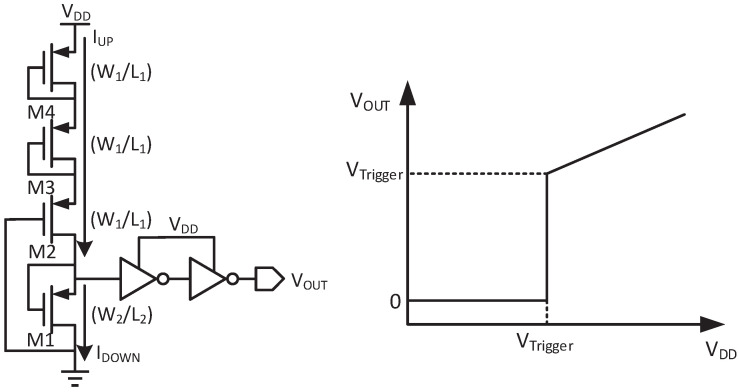
Schematic of the voltage detector and its waveform.

The voltage detector with its waveform is shown in Figure 11. When $V_{DD}$ reaches $V_{Trigger}$, $V_{OUT}$ turns high. The trigger voltage is determined by comparing $I_{UP}$ and $I_{DOWN}$. To reduce the power consumption, M1-M4 are working in the subthreshold region. According to [24], $I_{UP}$ is expressed as

(16) $$I_{UP}=\mu C_{ox}\frac{W_{1}}{L_{1}}e^{\frac{V_{DD}-V_{TH1}}{mV_{t}}},$$

where μ is the hole mobility, $C_{ox}$ is the gate oxide capacitance per area, *m* is a constant value of 1.1, $V_{t}$ is approximately 26 mV at room temperature(T = 300 K), and $V_{TH1}$ is the threshold voltage of M2–M4. $I_{DOWN}$ can be expressed as

(17) $$I_{DOWN}=\mu C_{ox}\frac{W_{2}}{L_{2}}e^{\frac{-V_{TH2}}{mV_{t}}},$$

where $V_{TH2}$ is the threshold voltage of M1. Once $I_{UP}$ is equal to $I_{DOWN}$, $V_{Trigger}$ turns high. If the threshold voltages of M1–M4 are equal and $V_{Trigger}$ is set to be 330 mV, the size ratio is derived as

(18) $$\frac{W_{2}/L_{2}}{W_{1}/L_{1}}=69.$$

However, if $V_{Trigger}$ increases to 1 V, the size ratio increases to 162,755, which is too large to implement. So a diode-connected PMOS can be added to M4, and its size is also $W_{1}$/$L_{1}$. Then under the same conditions, the size ratio decreases to 6250. As shown in Figure 11, the voltage detector can set the triggered voltage by adjusting the quantity of diode-connected PMOSs.

## 4. Circuit Verification

The proposed boost converter is implemented in a 0.18-μm standard CMOS process. The TEG is modeled as a DC power supply with an added series resistance of 5 Ω. A 100 μH inductor is used for the converter. To reduce the output ripples, the off-chip output capacitor is designed to be 10 μF.

**Figure 12:**
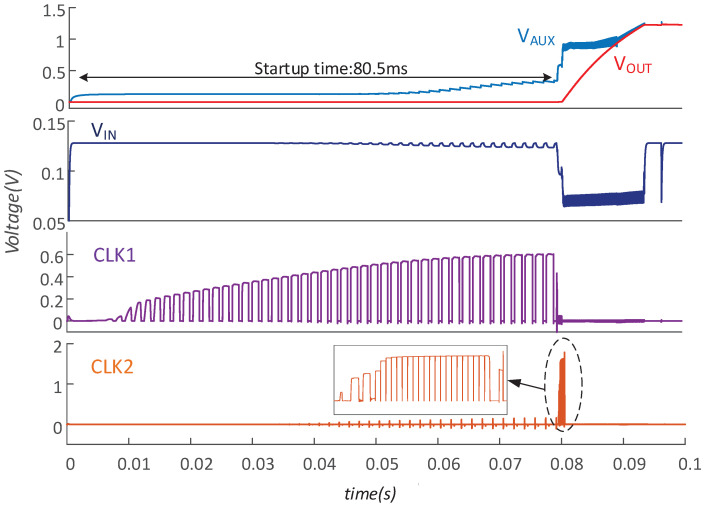
The waveform of startup process.

Figure 12 shows the coldstart process, which demonstrates that the converter starts with a minimum open-circuit voltage $V_{T}$ of 128 mV. Under this input condition, it takes 80.5 ms for $V_{AUX}$ to reach 600 mV. After self-starting, the inductor current is shared by two boost paths. Once $V_{AUX}$ ramps up to 1 V, $V_{AUX}$ is shorted with $V_{OUT}$ and the converter enters high-efficiency mode. Finally, the converter works temporarily and regulates $V_{OUT}$ at 1.2 V.

**Figure 13:**
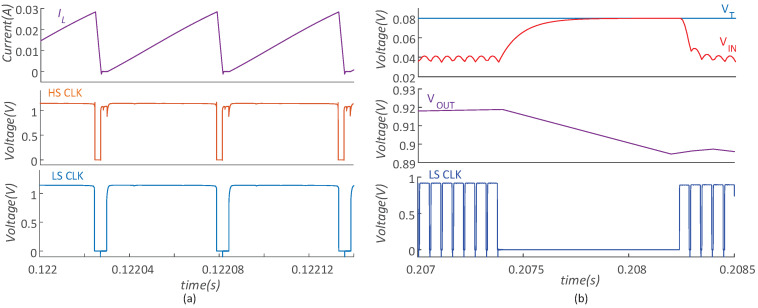
(**a**) The simulation results with $V_{T}$ of 128 mV with 600 μA load; (**b**) the sample operation.

Figure 13a shows the simulated results of the main clock and inductor current with 128 mV $V_{T}$. The frequency is 18.5 kHz and the on-time of LS is about 40 μs. Besides, the peak inductor current is 28 mA. The results show a negligible leakage in $I_{L}$, which indicates the proper current detection under a relatively heavy load condition with $I_{LOAD}$ = 600 μA. Figure 13b shows the sample phase in 80 mV $V_{T}$ with a 300 μA output current. Both LS and HS turn off, and thus, $V_{T}$ can be sampled. The sample time is 1 ms and $V_{OUT}$ decreases by 30 mV, which results in only 0.02% efficiency reduction in a sample period.

**Figure 14:**
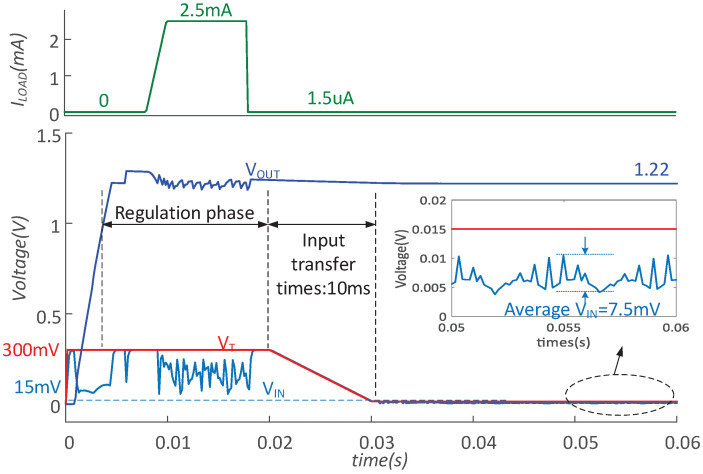
The simulated results with $V_{T}$ from 300 mV to 15 mV.

Figure 14 shows the system operation with MPPT when $V_{T}$ changes from 300 mV to 15 mV. During 10∼20 ms, the converter works intermittently at the open-circuit voltage of 300 mV, providing 2.5 mA output. After a 10 ms transition, the converter works with 15 mV $V_{T}$, providing 1.5 μA output. During 50∼60 ms, $V_{T}$ decreases to only 15 mV, while the minimum input voltage of proposed converter $V_{IN,MIN}$ is about 7.5 mV, which also means that the proposed MPPT still can efficiently work.

**Figure 15:**
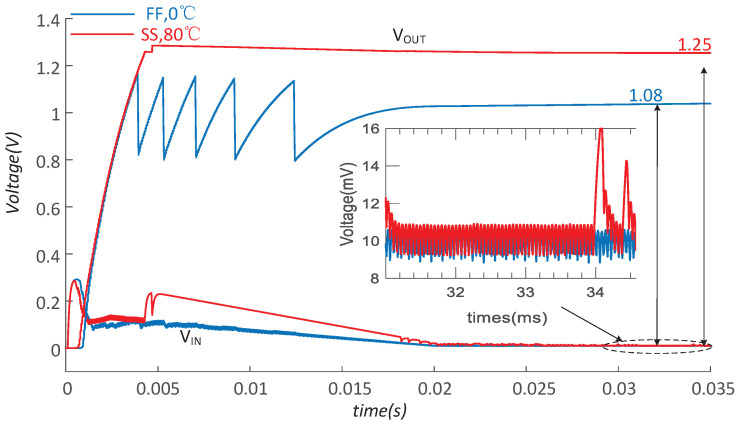
The simulation in different corners and temperature.

To verify the sensitivity of the proposed circuit to the process as well as temperature variations, and predict the performance after fabrication, the simulations with temperature ranging from 0 to 80 °C and different corners are carried out. Figure 15 shows the simulated output in FF corner at 0 °C and SS corner at 80 °C. The converter can normally work when $V_{T}$ changes from 300 mV to 20 mV and finally provides a 1.08 V and 1.25 V regulated output voltage, respectively. In the tracking phase, the input voltage $V_{IN}$ is about 10 mV, which indicates that the proposed MPPT can also efficiently and accurately work in different corners. Table 1 shows that the startup voltage increases to 130 mV in corner FF and 160 mV in the SS corner.

**Figure 16:**
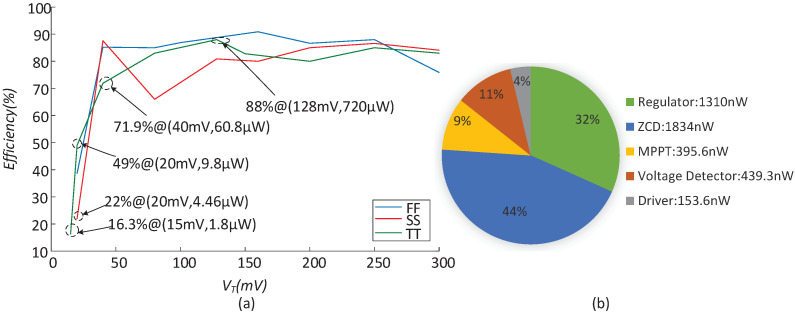
(**a**) The conversion efficiency and key output power in different corners in 27 °C; (**b**) the power consumption of each module.

Table 2 lists the tracking efficiency under the extreme temperatures and process corners. It can be observed that the tracking efficiency is greater than 93.6% when the input changes. Table 3 lists the conversion efficiency versus the light load current under different corners and temperatures. Meanwhile, it can be obtained from Figure 16a that the efficiency during the heavy load is not sensitive to the process. In the TT corner, the converter is able to sustain the operation at 15 mV $V_{T}$, which corresponds to only 1.8 μW output power. The efficiency is higher than 71.9% when $V_{T}$ changes from 40 mV to 300 mV. In particular, the peak efficiency is 88% when $V_{T}$ is 128 mV. Figure 16b shows the power consumption of each module when $V_{T}$ is 128 mV. The ZCD and regulator account for the heaviest proportion of 76%. The major power consumption of the regulator is attributed to the two dividing resistors.

Table 4 shows a performance comparison with the previous works. The proposed circuit provides a self-startup and regulated output with the maximum power of 3.78 mW, the minimum input voltage of 7.5 mV, and high efficiency of 88%.

## 5. Conclusions

A self-startup and single-inductor boost dc–dc converter for thermoelectric energy harvesting was presented and simulated in a standard 0.18 μm CMOS process in this paper. Based on the two stages with three-phase operation, the converter achieves self-startup at as low as 128 mV. Particular attention also was taken to obtain the accurate MPPT with as low as 7.5 mV input voltage by an adaptive coarse- and fine tuning. With the proposed MPPT and ZCD control, the converter has a peak efficiency of 88%, providing a maximum output power of 3.78 mW. These features together create the TEG-based harvester potential for self-powering wearable devices.

## Figures and Tables

**Table 1:** Startup voltage in different corners.

Corner	TT	FF	SS
Startup voltage (mV)	128	130	160

**Table 2:** Tracking efficiency under different corners and temperatures.

Corner	T	$V_{T}$	Tracking Efficiency
TT	0 °C	300 mV	99.7%
		15 mV	93.6%
	80 °C	300 mV	95%
		15 mV	98.9%
FF	0 °C	300 mV	99.1%
		20 mV	98%
	80 °C	300 mV	97.7%
		20 mV	99.8%
SS	0 °C	300 mV	99.1%
		20 mV	99%
	80 °C	300 mV	96.4%
		20 mV	99%

**Table 3:** Conversion efficiency versus light load current under different corners and temperatures.

$V_{T}$	$I_{\mathrm{LOAD}}$	Corner	T	Efficiency
300 mV	150 μA	TT	27 °C	47%
		FF	80 °C	44%
		SS	0 °C	43%
150 mV	100 μA	TT	27 °C	46%
		FF	80 °C	36.5%
		SS	0 °C	50.1%
20 mV	2 μA	TT	27 °C	12.9%
		FF	80 °C	13.9%
		SS	0 °C	7%

**Table 4:** Performance comparison.

	This Work	TCSII [25]	LSSC [26]	TCSII [27]	JSSC [15]	ISCAS [9]
Year	2022	2019	2021	2017	2016	2019
Technology (nm)	180	180	130	65	180	250
Inductor (μH)	100	N.A.	N.A.	47	33	33
$V_{T}$ (mV)	15–300	50–300	80–220	20–50	30–180	50–500
$V_{IN,MIN}$ (mV)	7.5	25	40	10	15	25
$V_{OUT}$ (V)	1.2	1–1.6	0.3	≥1	1.9	3
$R_{T}$ (Ω)	5	30	210	7	210	1
Peak $\eta_{Conv}$@$V_{IN}$	88%@64 mV	60%@60 mV	76.92%@80 mV	81%@25 mV	86.6%@40 mV	81%@150 mV
Self-startup voltage	128 mV	190 mV	300 mV	N.A.	N.A.	N.A.
Startup method	Coldtsart	Coldtsart	Coldtsart	External supplies	External precharge	External precharge
Maximum output power	3.78 mW	400 μW	N.A.	55 μW	30 μW	N.A.
MPPT	YES	YES	YES	NO	NO	YES
M or S result ^1^	Simulation	Measurement	Measurement	Measurement	Measurement	Simulation

^1^ Measurement or Simulation results.

## Data Availability

Not applicable.
